# Astroglia-Derived BDNF and MSK-1 Mediate Experience- and Diet-Dependent Synaptic Plasticity †

**DOI:** 10.3390/brainsci10070462

**Published:** 2020-07-18

**Authors:** Ulyana Lalo, Alexander Bogdanov, Guy W. Moss, Yuriy Pankratov

**Affiliations:** 1 School of Life Sciences, University of Warwick, Gibbet Hill Campus, Coventry CV4 7AL, UK; laloulya@yahoo.com; 2 School of Life Sciences, Immanuel Kant Baltic Federal University, 2 Universitetskaya str., Kaliningrad 236040, Russia; axebogdanov@gmail.com; 3 Department of Neuroscience, Physiology and Pharmacology, University College London, London WC1E 6BT, UK; g.moss@ucl.ac.uk

**Keywords:** aging, dendritic spines, synaptic strength, glia-neuron interactions, ion conductance microscopy, synaptic scaling, diet, enriched environment, GABA receptors, AMPA receptors, TrkB receptors, Arc/Arg3.1, calcium signalling

## Abstract

Experience- and diet-dependent regulation of synaptic plasticity can underlie beneficial effects of active lifestyle on the aging brain. Our previous results demonstrate a key role for brain-derived neurotrophic factor (BDNF) and MSK1 kinase in experience-related homeostatic synaptic scaling. Astroglia has been recently shown to release BDNF via a calcium-dependent mechanism. To elucidate a role for astroglia-derived BDNF in homeostatic synaptic plasticity in the aging brain, we explored the experience- and diet-related alterations of synaptic transmission and plasticity in transgenic mice with impairment of the BDNF/MSK1 pathway (MSK1 kinase dead knock-in mice, MSK1 KD) and impairment of glial exocytosis (dnSNARE mice). We found that prolonged tonic activation of astrocytes caused BDNF-dependent increase in the efficacy of excitatory synapses accompanied by enlargement of synaptic boutons. We also observed that exposure to environmental enrichment (EE) and caloric restriction (CR) enhanced the Ca^2+^ signalling in cortical astrocytes and strongly up-regulated the excitatory and down-regulated inhibitory synaptic currents in old wild-type mice, thus counterbalancing the impact of ageing on astroglial and synaptic signalling. The EE- and CR-induced up-scaling of excitatory synaptic transmission in neocortex was accompanied by the enhancement of long-term synaptic potentiation. Importantly, effects of EE and CR on synaptic transmission and plasticity was significantly reduced in the MSK1 KD and dnSNARE mice. Combined, our results suggest that astroglial release of BDNF is important for the homeostatic regulation of cortical synapses and beneficial effects of EE and CR on synaptic transmission and plasticity in aging brain.

## 1. Introduction

The ability of the brain to adapt to environmental and biochemical challenges during development and aging strongly depends on the capability of synapses to scale their strength in response to the activity of neighbouring neuronal networks [1,2,3]. Such form of responsiveness of synapses, conventionally termed as a homeostatic synaptic plasticity [2], has been implicated in the potential beneficial effects of exercise and active lifestyle on cognitive function [4,5,6,7,8]. Brain-derived neurotrophic factor (BDNF) has been shown to play important role in synaptic adaptation to both activity deprivation and environmental enrichment [1,9,10]. In particular, enhancement of BDNF production in response to environmental enrichment [1,3] can promote dendritic growth and enlargement of synaptic spines in rodents [11].

The role of BDNF in the homeostatic synaptic plasticity is mediated by complex cascade of molecular interactions starting from activation of tropomyosin receptor kinase B (TrkB) receptor and then involving a number of downstream proteins, the most important of which are cytoskeletal-associated protein Arc/Arg3.1 and mitogen and stress-activated kinase 1 (MSK1). We have shown previously that MSK1 protein kinase is instrumental for the BDNF-mediated homeostatic synaptic scaling and the effects of environmental enrichment (EE) on excitatory synapses in the hippocampus [9,12]. Since various environmental stimuli, such as physical activity or change in the diet, can enhance the release of BDNF [9,13,14], the BDNF/MSK1 pathway may underlie beneficial effects of exercise and caloric restriction (CR) on synaptic transmission and plasticity, in particular in the aging brain.

There is growing evidence that astroglia can play an important role in experience-related homeostatic regulation of synaptic networks. Firstly, astrocytes are responsive to the environmental enrichment (EE) and changes in the diet [15,16,17]. In particular, we have demonstrated that EE and CR can enhance astroglial Ca^2+^ signalling [15]. Second, astrocytes, which control chemical environment of synapses and form an interface between neurons and brain vasculature [17,18,19] are ideally positioned to couple the changes in physical activity and diet to the biochemical changes in neurons. This could be done by two alternative but not mutually exclusive mechanisms—regulation of metabolic support of neurons and modulation of synaptic plasticity by releasing various gliotransmitters, such as ATP and D-Serine [15,19,20,21,22]. Importantly, astroglia can serve as an important source of BDNF by releasing it via Ca^2+^-dependent exocytosis [23,24]. Thus, one could suggest that astroglia-derived BDNF could underlie the effects of exercise and diet on homeostatic synaptic scaling and synaptic plasticity. 

To verify this hypothesis, we explored effects of tonic activation of astrocytes in situ and in vivo (via environmental stimuli) on synaptic scaling in transgenic mice with impairment of the BDNF/MSK1 pathway (MSK1 kinase dead knock-in mice) and impairment of glial exocytosis (dnSNARE mice). We also explored the putative roles for these pathways in aging-related changes in synaptic dynamics and long-term synaptic plasticity. The present article is updated and extended version of our previous publication [25].

## 2. Material and Methods

All animal work was carried out in accordance with UK legislation and the ‘3R’ strategy; research did not involve non-human primates. This project was approved by the University of Warwick Animal Welfare and Ethical Review Body (AWERB), approval number G13-19, and regulated under the auspices of the UK Home Office Animals (Scientific Procedures) Act licenses P1D8E11D6 and I3EBF4DB9. Experiments were carried out in the astrocytes and neurons of the hippocampus and somatosensory cortex of dnSNARE transgenic mice [26,27], their wild-type littermates (WT), and transgenic mice with MSK1 kinase dead knock-in (MSK1 KD mice) [9]; the MSK1 KD mice had the same genetic background as the dnSNARE mice (C57/Bl6). We used mice of two age groups, 6–12 (average 7.8) weeks and 9–15 (average 12.7) months which were kept either under standard housing conditions (SH) vs. animals exposed to the EE from birth [9], including ad libitum access to the running wheel, or kept on mild calorie restriction diet for 4 to 6 weeks. According to the protocol of caloric restriction, food intake was reduced by 10–20% and was individually regulated to maintain the body weight loss within 10–15%.

### 2.1. Slice and Cell Preparation

Mice were anaesthetized by halothane and then decapitated, and their brains were removed rapidly after decapitation and placed into ice-cold physiological saline containing (mM): NaCl 130, KCl 3, CaCl_2_ 0.5, MgCl_2_ 2.5, NaH_2_PO_4_ 1, NaHCO_3_ 25, glucose 15, pH of 7.4 gassed with 95% O_2_–5% CO_2_. Transverse slices (260 µm) were cut at 4 °C and then placed in physiological saline containing (mM): NaCl 130, KCl 3, CaCl_2_ 2.5, MgCl_2_ 1, NaH_2_PO_4_ 1, NaHCO_3_ 22, glucose 15, pH of 7.4, gassed with 95% O_2_–5% CO_2_, and kept for 1–5 h prior to cell isolation and recording. 

Astrocytes were identified by their morphology under DIC observation, EGFP fluorescence (astrocytes from dnSNARE mice) or staining with sulforhodamine 101 (astrocytes from WT and MSK1 KD mice). After recording, the identification of astrocyte was confirmed via functional properties (high potassium conductance, low input resistance, strong activity of glutamate transporters) as described previously [27,28,29]. 

Hippocampal cultures were prepared from postnatal day 0 or 1 wild-type (WT) and MKS1 KD mice. After decapitation, hippocampi were extracted from the brain at 4 °C, and hippocampal cells were dissociated by enzymatic digestion with trypsin and mechanical dissociation through a Gilson pipette. Then, cells were plated onto 22-mm glass coverslips coated with poly-L-lysine hydrobromide (0.5 mg/mL, Sigma-Aldrich, Gillingham, Dorset, UK) at a density of approximately 100,000 cells per dish. The plating medium contained Neurobasal-A medium (Gibco, Themo Fisher Scientific, UK) supplemented with Gentamicin (Gibco), 1% L-Glutamine (Gibco), 2% B27 (Gibco) and 5% horse serum (Gibco). One day after plating, the plating medium was changed for horse serum free feeding medium. The cultures were then maintained in in a 95% O_2_/5% CO_2_–humidified incubator and fed every 5–6 days. All experiments were performed using cells at 12–16 days in vitro (DIV). The resulting hippocampal cell cultures contained both neurons and astrocytes as were confirmed by morphological and electrophysiological characterization and immunostaining with neuronal (PSD-95) and astrocytic markers (GFAP and S100β).

### 2.2. Electrophysiological Recordings

Whole-cell voltage-clamp recordings from cortical neurones and astrocytes cells were performed using glass patch-pipettes (4–5 MΩ) filled with intracellular solution (in mM): 110 CsCl, 10 NaCl, 10 HEPES, 5 MgATP, 1 D-Serine, 0.1 EGTA, pH 7.35; Transmembrane currents were monitored using an MultiClamp 700 B patch-clamp amplifier (Axon Instruments, Union City, CA, USA) filtered at 2 kHz and digitized at 4 kHz. Experiments were controlled by Digidata1440 A data acquisition board (Axon Instruments) and WinWCP software (Strathclyde University, Edinburgh, UK); data were analysed by self-designed software. Liquid junction potentials were compensated with the patch-clamp amplifier. The series and input resistances were respectively 5–7 MΩ and 600–1100 MΩ; both series and input resistance varied by less than 20% in the cells accepted for analysis. When spontaneous synaptic currents were recorded, the inhibitor of P2X purinoreceptors PPADS (10 µM) was added to the extracellular solution to prevent a possible interference of from small fraction of purinergic excitatory spontaneous currents [27,30] on amplitude distribution of mEPSCs.

Field excitatory postsynaptic potentials (fEPSPs) were measured via a glass micropipette filled with extracellular solution (0.5–1 MΩ resistance) placed in neocortical layer II/III. The fEPSPs were evoked by the stimulation of neuronal afferents descending from layers IV-V. For activation of synaptic inputs, axons originating from layer IV-VI neurons were stimulated with a bipolar coaxial electrode (WPI-Europe, Hitchin, UK) placed in layer V close to the layer IV border, approximately opposite the site of recording; stimulus duration was 300 µs. The stimulus magnitude was set 3–4 times higher than the minimal stimulus necessary to elicit a response in layer II pyramidal neurons [28,29,31].

The long-term potentiation (LTP) was induced by 50 trains of high-frequency theta-burst stimulation (HFS-trains); each train (100 ms-long) consisted of 10 pulses stimulated at 100 Hz, trains were delivered with 200 ms intervals, and every 10 trains were separated by 10-s-long intervals. 

### 2.3. High-Resolution Scanning of Synaptic Boutons 

The morphology of synaptic boutons was imaged in the neuronal cultures using the super-resolution hopping-probe ion conductance microscopy, an advanced version of scanning ion conductance microscopy (SICM) [32]. The identification of synaptic boutons and SICM 3D topographical imaging were carried out using a custom-modified SICM scanner ICNano (Ionosope, London, UK) as described previously [32]. Briefly, the sample was positioned in the X–Y directions with nanopositioning stage (Physik Instrumente, Karlsruhe, Germany) and the scanning pipette was positioned in z-direction with piezoelectric actuator. The fine-tipped scanning nanopipettes were pulled from borosilicate glass (OD 1 mm, ID 0.5 mm) with a horizontal laser puller P-2000 (Sutter Instruments, Novato, CA, USA); pipette resistance was in the range of 80–100 MOhm, corresponding to the estimated tip diameter of 90–120 nm. Nanopipettes were held in the voltage-clamp mode with an Axopatch 200 B patch-clamp amplifier (Axon Instruments); an amplifier head-stage was mounted on the z-scanning head. The amplifier output signal was monitored by the SICM electronics which simultaneously controlled the sample and pipette positioning.

The scan system was mounted onto inverted microscope Nikon TE2000-U (Nikon Instruments, Kingston, UK) equipped with epifluorescence illumination. The synapses were pre-stained with fluorescent synaptic marker.

FM1-43 using for 15 min-long application of 3 µM of dye followed by 15 min washout. The FM1-43 fluorescence was imaged 100 × 1.3 NA oil-immersion objective and EM-CCD camera (Andor iXon3, Andor Technology, Belfast, UK). Synaptic boutons were identified by matching the tentative boutons in topography and FM1-43 fluorescence [32]. The raw SICM data was processed using the Gwyddion 5.0 microscopy analysis software (Czech Metrology Institute, Brno, Czech Republic) [33] (http://gwyddion.net/). After constructing a 3D topographical image of identified synaptic varicosities their maximal span in X–Y–Z dimensions (Δx, Δy, and Δz) has been determined and the effective size have been calculated as square root of (Δx^2^ + Δy^2^ + Δz^2^) quotient. The synaptic varicosity volume has been estimated as volume of an ellipsoid of Δx, Δy, and Δz dimensions.

### 2.4. Multi-Photon Fluorescent Ca^2+^-Imaging in Astrocytes

To monitor the concentration of cytoplasmic free Ca^2+^ ([Ca^2+^]_i_) in situ, astrocytes of neocortical slices were loaded via 30 min incubation with 1 µM of Rhod-2AM (dnSNARE mice) or Oregon Green Bapta-2AM and sulphorhodamine 101 (wild-type and MSK1 KD mice) at 33 °C. Two-photon images of neurons and astrocytes were acquired at 5 Hz frame-rate using a Zeiss LSM-7MP multi-photon microscope coupled to a SpectraPhysics MaiTai (SpectraPhysics, Santa Clara CA, USA) pulsing laser; experiments were controlled by ZEN LSM software (Carl Zeiss, Jena, Germany). Images were further analysed offline using ZEN LSM (Carl Zeiss) and ImageJ 1.52 (NIH) software. The [Ca^2+^]_i_ levels were expressed as ΔF/F ratio averaged over a region of interest (ROI). For analysis of spontaneous Ca^2+^-transients in astrocytes, three ROIs located over peripheral astrocytic processes and 1 ROI located over the soma were chosen. Overall Ca^2+^-response to receptors agonists or synaptic stimulation was quantified using an ROI covering the whole cell image. To quantify the net response of a cell to stimulation, Ca^2+^-signal was integrated over 3 min immediately after agonist application, averaged over the whole cell image and normalized to the baseline integral Ca^2+^-signal.

### 2.5. Data Analysis 

All data are presented as mean ± standard deviation (SD), and the statistical significance of differences between data groups was tested by two-tailed unpaired *t*-test, unless indicated otherwise. For all cases of statistical significance reported, the statistical power of the test was 0.8–0.9. Each neocortical slice was used only for one experiment (e.g., fluorescent recordings in single astrocyte or single LTP induction experiment). The number of experiments/cells reported is therefore equal to the number of slices used. The experimental protocols were allocated randomly so the data in any group were drawn from at least from three animals and typically from 4–10 mice. The average ratio of experimental unit per animal was 1.28 for the LTP experiments and 1.55 whole-cell recordings and fluorescent Ca^2+^-measurements. The spontaneous transmembrane currents recorded in neurons were analysed off-line using methods described previously [27,31]. The amplitude distributions of spontaneous and evoked currents were analysed with the aid of probability density functions and likelihood maximization techniques; all histograms shown were calculated as probability density functions. The amplitude distributions were fitted with either multi-quantal binomial model or bi-modal function consisting of two Gaussians with variable peak location, width, and amplitude. Parameters of models were fit using likelihood maximization routine. 

## 3. Results

### 3.1. Astroglia-Induced Homeostatic Synaptic Scaling in Cultured Neurons

A typical experimental approach in studies of homeostatic synaptic scaling anticipates chronic inhibition or enhancement of neuronal firing in primary cultures using 12–24 hour-long incubation with TTX or picrotoxin [9,10]. To explore the specific role for astrocytes, we used similar paradigm but, instead of neurons, we selectively enhanced astroglial Ca^2+^ signalling with TFLLR peptide, an agonist of PAR-1 receptors. The efficiency and specificity of PAR-1 receptor-mediated activation of astrocytes and lack of non-specific effects of TFLLR in neurons have been verified previously [27]. 

**Figure 1:**
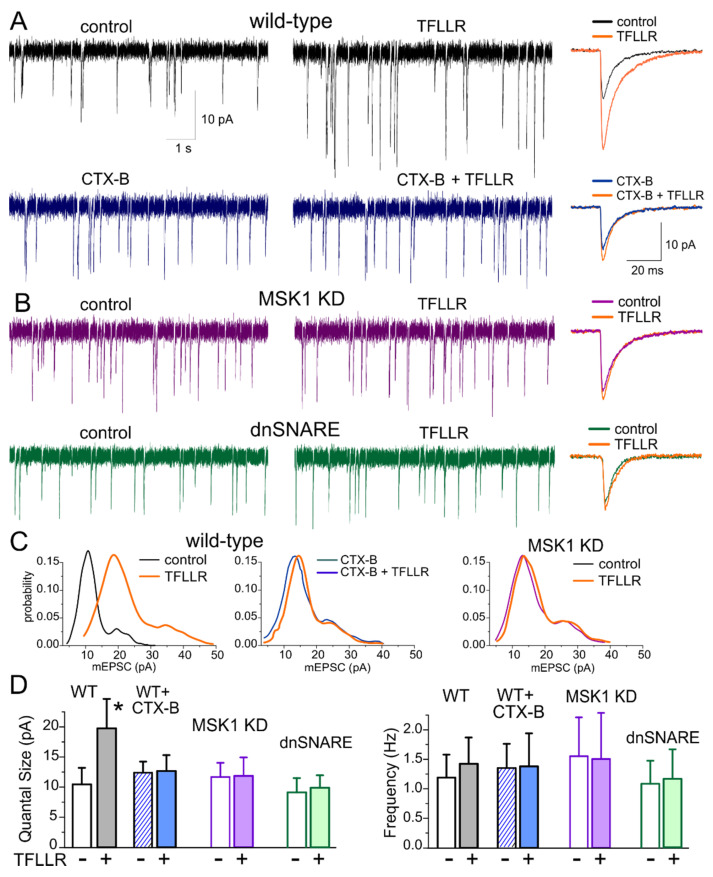
MSK1 and BDNF mediate astroglia-induced synaptic scaling in hippocampal neurons. Synaptic scaling was induced in the hippocampal pyramidal neurons in culture via incubation with selective astroglia activator TFLLR. (**A**) The representative mEPSCs recorded in the wild-type neurons in the control media and after 24 hour-incubation with either 3 µM TFLLR alone or TrkB inhibitor Cyclotraxin B (10 nM) alone or TFLLR and Cyclotraxin B together. Synaptic currents were recorded at membrane potential of −80 mV in the presence of picrotoxin (100 µM), TTX (1 µM) and PPADS (pyridoxalphosphate-6-azophenyl-disulfonic acid, 10 µM). (**B**) The mEPSCs recorded in the neurons of MSK1 KD and dnSNARE mice in the control and after incubation with TFLLR. The insets on the right show the corresponding average mEPSC waveforms. (**C**) The mEPSC amplitude distributions recorded in the same neurons as in (**A**,**B**). Note that the unitary quantal size, indicated by the position of the main peak in the amplitude distribution, undergoes a considerable increase, suggesting a postsynaptic effect of exposure to TFLLR in the wild-type but not MSK1 KD neurons or in the WT neurons in the presence of BDNF receptor blocker. (**D**) Pooled data on the quantal size and average frequency of mEPSCs recoded as described above. Data show mean ± SD for 9–12 neurons from three to four primary neuronal cultures. Note the significant increase (*p* < 0.005, unpaired *t*-test) in the quantal mEPSC amplitude in wild-type neurones after incubation with TFLLR. The lack of significant changes in the mEPSC frequency supports a postsynaptic origin of the effect. In contrast, activation of astrocytes with TFLLR did not cause significant alterations in mEPSCs in MSK KD1 neurons or WT neurons in the presence of BDNF receptor blocker.

We recorded the miniature excitatory postsynaptic currents (mEPSCs) in the hippocampal pyramidal neuron in presence of picrotoxin (100 µM) and tetrodotoxin (1 µM). Enhancement of astrocytic signalling by 24 hour-long incubation of primary hippocampal cultures with 3 µM TFLLR led to the significant up-scaling of glutamatergic mEPSCs (Figure 1A). The average mEPSC amplitude increased in the wild-type neurons from 14.01 ± 2.77 pA (*n* = 11) to 26.7 ± 6.55 pA (*n* = 9, *p* < 0.01).

To verify the crucial role for BDNF in the observed astroglia-induced synaptic up-scaling, we incubated wild-type cultured neurones in TFLLR together with selective and potent Trk-B receptor inhibitor Cyclotraxin-B (CTX-B) [34]. Blocking the BDNF action with 10 nM of CTX-B prevented the TFFLR-induced enhancement of mEPSCs (Figure 1A). Furthermore, the 24-hour-long application of TFLLR did not cause marked changes in synaptic currents in the cell cultures derived from MSK1 KD mice (Figure 1B,D), strongly supporting the crucial role for BDNF-MSK1 pathway in the glia-induced synaptic scaling.

Analysis of quantal behaviour of mEPSCs, illustrated in Figure 1C, revealed that TFLLR-induced enhancement of average amplitude resulted from the increase in the quantal size of synaptic response (manifested as a main peak of amplitude distribution). The incubation with TFLLR (in the absence of CTX-B) caused the significant increase in the quantal size of mEPSCs only in the neurons of wild-type but not MSK1 KD or dnSNARE mice (Figure 1A,C)**.** At the same time, incubation with TFLLR did not lead to marked changes in the mEPSCs frequency (Figure 1C). These results demonstrate that glia-induced BDNF-dependent homeostatic synaptic scaling occurs mainly via postsynaptic mechanisms, similarly to previously reported forms of homeostatic synaptic plasticity [9].

### 3.2. Astroglia-Induced Homeostatic Changes in Synaptic Morphology

Very often, up-regulation of excitatory synaptic transmission at postsynaptic locus occurs via increase in the average number of AMPA receptors per synapse and, correspondingly, increase in the size of dendritic spines. The changes in synaptic morphology have been reported to be mediated by BDNF which can induce the expression of Arc/Arg3.1 gene [9,35,36]. Thus, one might expect that astroglia-induced synaptic scaling involved BNDF-dependent enlargement of dendritic spines on pyramidal neurons. 

To test this hypothesis, we assessed the TFFLR-induced alterations of the size of postsynaptic densities using scanning ionic conductance microscopy (SICM). This technique is based on a decrease in the ionic current passing through a glass microelectrode in the close proximity of cells (on any other obstacles)—a phenomenon familiar to any electrophysiologist. The SICM method allows morphological imaging of live cells at nanoscale resolution and adequately map the shape and size of small subcellular structures, such as dendrites, axons and synaptic boutons [32]. This technique has been previously used in various studies of synaptic networks of cultured hippocampal neurons and mechano-sensory stereocilia of cochlear hair cells [32,37].

**Figure 2:**
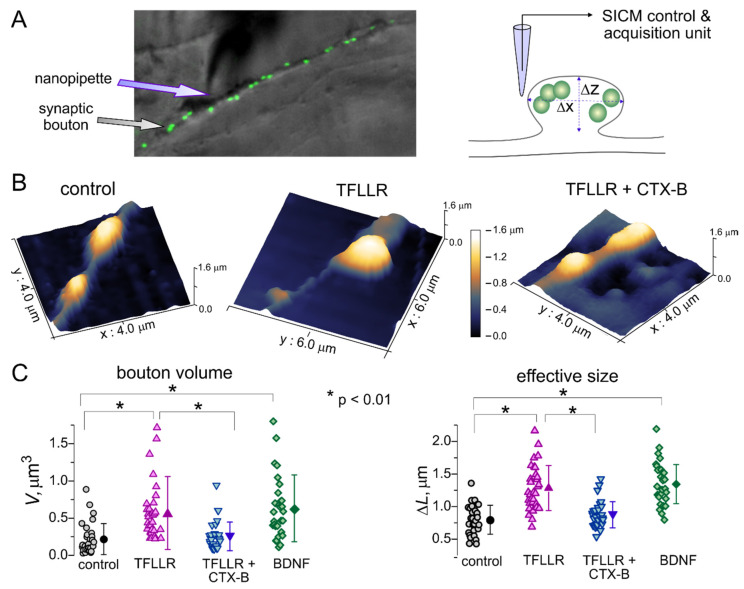
Astroglia and BDNF regulate synaptic size. (**A**) The principle of tomographic imaging of synaptic boutons using the high-resolution hopping probe scanning ion conductance microscopy (SICM). Synapses of live hippocampal neurons were stained with synaptic vesicle marker FM1-43. (**B**) High-resolution SICM 3D images of hippocampal synapses of wild-type neurons in control and after 24 hour-incubation with 3 µM TFLLR or TFLLR and Cyclotraxin B (10 nM). The X-, Y- and Z-scales are indicated at the images, the Z-scale is highlighted as pseudo-color (same Z-scale for all three images). (**C**) The pooled data on the size and volume of synaptic boutons of wild-type neurons in control and after 24 hour-incubation with TFLLR, TFLLR and Cyclotraxin B (10 nM), and BDNF (100 ng/mL). Open symbols indicate individual boutons, the closed symbols show mean ± SD (*n* = 30–35 boutons from three preparations). Note the significant increase in the size and volume of synapses after incubation with TFLLR or BDNF.

Putative effects of astroglia-derived BDNF on synaptic morphology (Figure 2A) were examined in the live neurons of wild-type mice hippocampal cultures incubated with TFLLR alone, TFLLR with inhibitor of TrkB receptors or BDNF (100 ng/mL). Prior to the SICM imaging, functional synapses on hippocampal neurons were identified by labelling with FM1-43, an activity-dependent marker of synaptic vesicles, (Figure 2A). The SICM mapping in the vicinity of FM1-4 spots revealed the varicosities which size and shape were consistent with a geometry expected of synaptic boutons. We targeted the fluorescently labelled varicosities located on the distal dendrites to increase probability to encounter excitatory synapses. Upon 3D mapping of identified synaptic boutons (Figure 2B), their volume and effective size were evaluated as described in the Methods section (Figure 2C). 

**Figure 3:**
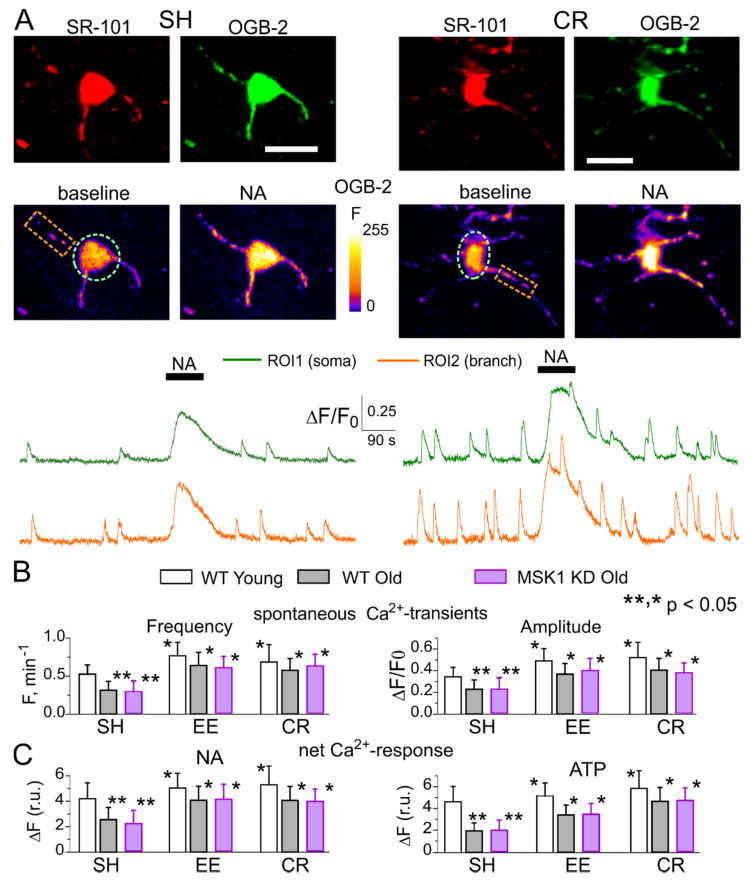
Age- and experience-related changes in the astrocytic Ca^2+^ signalling. Astroglial Ca^2+^ signalling was evaluated in the neocortex of 6–12 week-old (young) and 9–15 month-old mice (old). The MSK1 KD and their wild-type littermates were kept either in standard housing (SH) or exposed to environmental enrichment (EE) or caloric restriction (CR) as described in the *Methods*. (**A**) Representative multi-photon images of astrocytes of old MSK1 KD mouse pre-loaded with OregonGreen BAPTA-2 AM and stained with fluorescent astroglial marker SR101 and presudo-colour images of OGB-2 fluorescence recorded before and after application of noradrenaline (NA, 1 µM). Graphs below show the time course of OGB-2 fluorescence averaged over regions indicated in the fluorescent images. Note the increase in the amplitude of spontaneous in the Ca^2+^ -elevations and response to NA. (**B**) The pooled data on peak amplitude and frequency of the baseline spontaneous Ca^2+^- transients recorded in astrocytes of WT and MSK1 KD mice of different age and treatment groups. Number and size of spontaneous events were pooled for the whole cell image. (**C**) The pooled data on the net cell responses to application of 1 µM noradrenaline and 10 µM ATP. Data in the panels (**B**,**C**) are shown as mean ± SD for the 6–12 astrocytes from 3–4 animals. Asterisks (*, **) correspondingly indicate statistical significance of the effect of EE- or CR-treatment (as compared to SH) and difference between the old and young mice of the same treatment group. Note the significant increase in the spontaneous and evoked Ca^2+^ signalling in astrocytes of mice exposed to EE and CR and the lack of difference in the Ca^2+^ signalling in the WT and MSK1 KD.

We observed the considerable enlargement of synaptic boutons after 24-hour incubation of neuronal cultures with TFLLR (Figure 2). Under control conditions, the average size of synaptic varicosities increased from 0.798 ± 0.223 µm to 1.248 ± 0.345 µm (*n* = 31, *p* < 0.01). Correspondingly, the average volume of synaptic boutons showed >2.5-fold increase, from 0.218 ± 0.208 µm^3^ to 0.569 ± 0.489 µm^3^ (*p* < 0.01). Consistent with our observations of astroglia-induced enhancement of synaptic currents, the effect of TFLLR was effectively inhibited by Cyclotraxin-B (average size increased just by 10%) and reproduced by incubation with BDNF (Figure 3C). 

Taken together, our results on alterations of synaptic strength and morphology demonstrate that activation of astrocytes can engage the BDNF- and MSK1-dependent molecular cascades that are instrumental for the homeostatic scaling of synaptic transmission. 

### 3.3. Astrocytes Participate in Homeostatic Plasticity In Vivo

Previously, we demonstrated that BDNF-mediated homeostatic synaptic scaling can underlie effects of environmental enrichment on synaptic transmission [9]. Also, an increase in the BDNF level has been implicated into beneficial effects of a low-calorie diet on brain longevity and cognitive health [13,38]. Since environmental enrichment and caloric restriction can enhance Ca^2+^ signalling in the neocortical astrocytes [15], it is conceivable that astroglial Ca^2+^-dependent release of BDNF plays a key role in beneficial effects of EE and CR on synaptic transmission in aging brain. To test this hypothesis, we used a behavioural model of experience-dependent synaptic plasticity and compared effects of environmental enrichment and caloric restriction on synaptic transmission in the neocortex of the MSK1 KD and dnSNARE mice with their wild-type counterparts. 

First, we had to verify that EE and CR could up-regulate Ca^2+^ signalling in the neocortical astrocytes of MSK1 KD mice. As we did previously in the wild-type and dnSNARE mice [27,28,29], we monitored spontaneous cytosolic Ca^2+^-transients in the branches and soma of neocortical astrocytes of 6–12-week-old (young adults) and 9–15-month-old (old) mice with the aid of multi-photon fluorescent microscopy (Figure 3). We did not find any significant difference in the astroglial Ca^2+^ signalling in the old wild-type and old MSK1 KD mice (Figure 3). Furthermore, we observed similar age- and experience-related changes in astroglial signalling in all strains tested; the amplitude and frequency of spontaneous astrocytic Ca^2+^-transients in the old mice of standard housing were considerably lower than those ones measured in the young mice. Most importantly, EE and CR increased the spontaneous astroglial Ca^2+^ signalling in the old WT and MSK1 KD mice to the similar extent (Figure 3A,B). We also evaluated the astrocytic Ca^2+^-transients activated via noradrenaline (NA) and ATP receptors because release of these neurotransmitters can increase during enhanced neuronal and physical activity [31,39,40]. Receptors to these transmitters, in particular α1AR and P2Y, were reported to bring substantial contribution to astrocytic Ca^2+^ signalling and glia-neuron interactions [28,31,39,40]. In young mice, the amplitudes of astrocytic responses to NA (3 µM) and ATP (30 µM) underwent a moderate increase after exposure to EE and CR. In old animals, however, EE and CR caused the significant enhancement of astroglial adrenergic and purinergic signalling (Figure 3C). 

The above experiments have not revealed any significant difference in the Ca^2+^ signalling in astrocytes of old MSK1 KD vs. old wild-type mice, including the effects of EE and CR (Figure 3C). We have previously reported that astroglial Ca^2+^ signalling is not altered in the dnSNARE mice either [15,27,29]. These results can be very useful for unequivocal interpretation of EE- and CR-induced alterations in synaptic transmission. First, one can expect an EE- and CR-induced enhancement of astroglial Ca^2+^ signalling and astrocyte-neuron communication. Second, any putative difference in the impact of EE and CR on synaptic transmission in the dnSNARE and MSK1 KD mice should be attributed to impairment of downstream signalling cascades, correspondingly astroglial exocytosis and BDNF-mediated regulation of synaptic strength rather than the deficit in astroglial Ca^2+^-elevation. 

**Figure 4:**
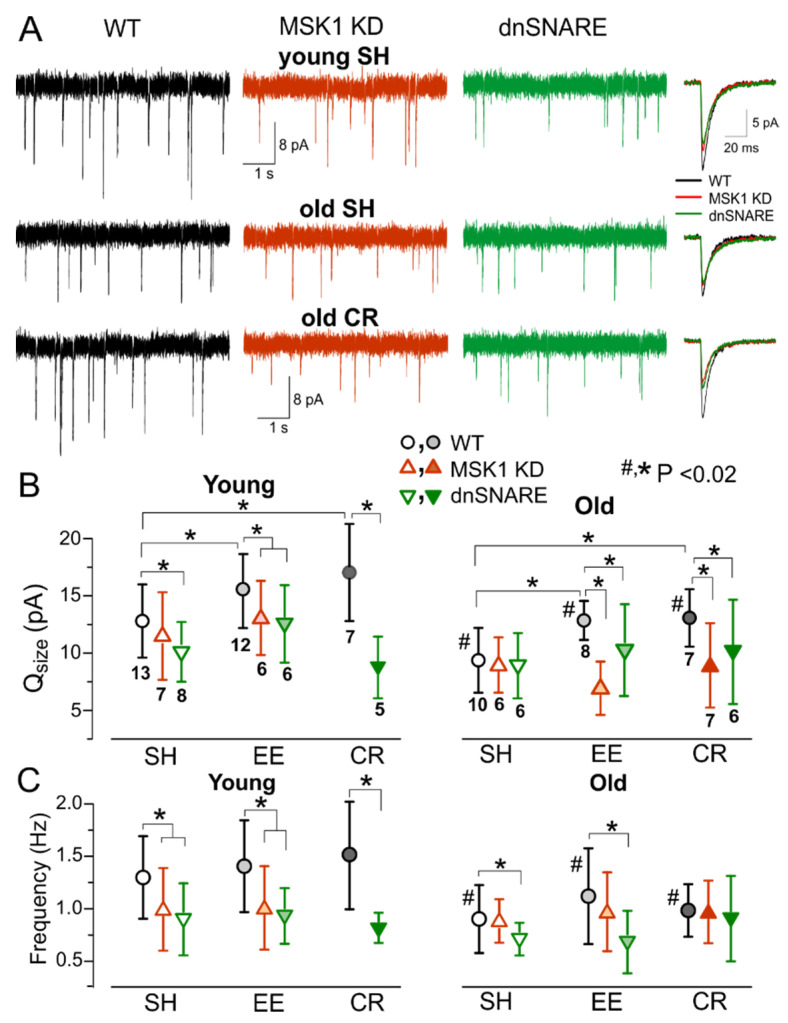
The experience-related alterations in the excitatory synaptic transmission. The AMPA receptor-mediated miniature spontaneous synaptic currents (mEPSCs) were recorded in the neocortical layer 2/3 pyramidal neurons at −80 mV in presence of 100 µM picrotoxin, 1 µM TTX and 10 µM PPADS. (**A**) representative whole-cell currents recorded in neurons of young (top row) and old SH (middle row) and EE (bottom row) WT, MSK1 KD, and dnSNARE mice (left, middle, and right columns, respectively). The inserts on the right shows average mEPSCs waveforms. Note the significant decrease in the mEPSC amplitude in the old WT mice of standard housing and up-regulation of mEPSCs in the EE mice. The effect of EE is impaired in the MSK1 KD and dnSNARE mice. (**B**,**C**) Pooled data on the quantal size (**B**) and frequency (**C**) of mEPSCs in the mice of different age and experience groups. Data are shown as mean ± SD for the number of neurons indicated in (**B**). The statistical significance (2-population unpaired *t*-test) for the difference between young and old mice of same genotype and experience is indicated by (#) symbols; asterisks (*) indicate statistically significance of the difference between different genotype and experience groups as indicated.

To explore the experience-related homeostatic plasticity in the neocortex, we carried out ex-vivo recordings of excitatory synaptic currents in neocortical neurons of mice exposed to EE or CR. The AMPA receptor-mediated mEPSCs were measured in the pyramidal neurons in the presence of picrotoxin (100 µM) and PPADS (10 µM). In the young wild-type mice, exposure to EE increased the average amplitude of mEPSCs to 18.6 ± 6.04 pA (*n* = 12), as compared to 14.3 ± 5.97 (*n* = 13) in their SH counterparts. The amplitude of mEPSCs was lower in the older WT mice but exhibited much larger EE-induced up-scaling (Figure 4A,B). The average mEPSC amplitude in the old SH mice increased from 10.86 ± 3.55 pA (SH, *n* = 10) to 14.34 ± 3.47 pA (EE, *n* = 8). Both quantal size and frequency of mEPSCs were reduced in the old mice, indicating that aging can affect synaptic transmission at pre- and post-synaptic loci. In contrast, EE-induced increase in average amplitude of mEPSCs was accompanied only by the significant increase in their quantal size whereas the frequency of mEPSCs did not undergo considerable changes (Figure 4C), so this effect had a postsynaptic origin. Similarly to EE, the exposure to CR led to increase in the average amplitude and quantal size of mEPSCs in the WT mice, CR-induced up-scaling was more prominent in older mice (Figure 4A–C). 

In stark contrast to the WT mice, mEPSCs recorded in the neurons of MSK1 KD mice underwent the age-related decline but did not exhibit significant EE- or CR-induced increase in the average amplitude or quantal size (Figure 4A,B) supporting the importance of MSK1 for the homeostatic plasticity. The mEPSCs recorded in the dnSNARE mice exhibited only modest EE- and CR-induced increase in the average mEPSCs amplitude. The difference in the quantal size of mEPSCs between WT and dnSNARE mice was statistically significant only for the EE-exposed animals of younger age (*p* < 0.05). The incomplete inhibition of EE-induced homeostatic up-scaling of mEPSCs in the young dnSNARE mice could be attributed to the mosaic (50–60% of cells) expression of dnSNARE transgene across astroglial cell population [26] and therefore incomplete loss of glial exocytosis. Also, neuronal release of BDNF could not be excluded. The dependence of EE- and CR-induced up-scaling of excitatory synaptic currents in the neocortex on the MSK1-signalling pathway and astrocytic exocytosis closely agrees with our result obtained in cell cultures (Figure 1 and Figure 2). Our present data also agree with previous results on key role of BDNF/MSK1 pathway for EE-induced synaptic scaling in hippocampal neurons. Combined together, our in vitro and ex vivo data strongly support the crucial importance of astroglial release of BDNF for experience-related plasticity of excitatory synaptic transmission.

**Figure 5:**
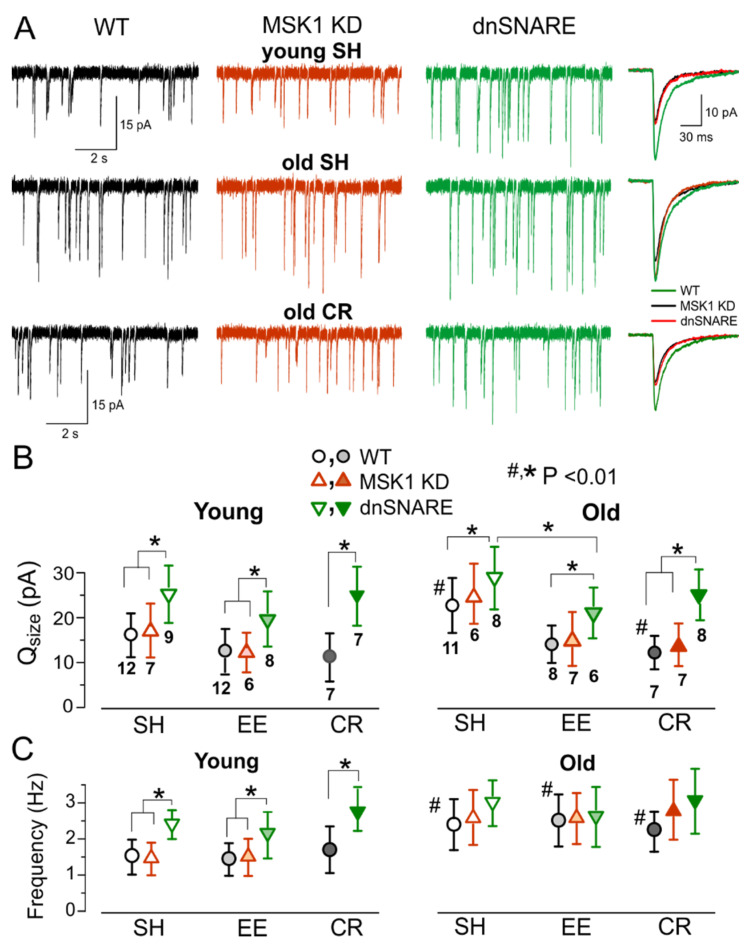
The experience-related alterations in the inhibitory synaptic transmission. The GABA receptor-mediated miniature inhibitory synaptic currents (mIPSCs) were recorded in the neocortical layer 2/3 pyramidal neurons at −80 mV in presence of 30 µM DNQX, 1 µM TTX and 10 µM PPADS. (**A**) the representative whole-cell currents recorded in the neurones of young (top row) and old SH (middle row) and EE (bottom row) WT, MSK1 KD, and dnSNARE mice (left, middle, and right columns, respectively). The inserts on the right shows an average mIPSCs waveforms. Note the significant increase in the mIPSC amplitude in the old WT mice of standard housing and down-regulation of mIPSCs in the EE mice. The effect of EE is impaired in the MSK1 KD and dnSNARE mice. (**B**,**C**) Pooled data on the quantal size (**B**) and frequency (**C**) of mIPSCs in the mice of different age and experience groups. Data are shown as mean ± SD for the number of neurons indicated in (**B**). The statistical significance (2-population unpaired *t*-test) for the difference between young and old mice of same genotype and experience is indicated by (#) symbols; asterisks (*) indicate statistically significance of the difference between different genotype and experience groups as indicated.

Following the similar experimental paradigm, we explored the experience-dependent plasticity of inhibitory synaptic signalling. The GABA receptor-mediated mIPSCs were recorded in neocortical neurons at membrane potential of −80 mV in the presence of DNQX and PPADS (Figure 5). We observed considerable age- and environment-related alterations in the inhibitory GABAergic transmission which exhibited trends quite different from changes in the glutamatergic signalling. First, inhibitory currents were significantly upregulated in the neurons of older WT and MSK1 KD mice kept under standard housing conditions mIPSCs. In the dnSNARE mice, both quantal size and frequency of mIPSCs were much higher than in their WT-littermates in both age groups (Figure 5A,B); this result goes in line with our previous observations [19]. Second, exposure of the wild-type mice to EE and CR efficiently down-regulated the inhibitory synaptic signalling, especially in the neurons of older mice (Figure 5A,B). In contrast to mEPSCs, the EE- and CR-induced downscaling of mIPSCs was abolished in the dnSNARE but not in the MSK1 KD mice suggesting that BDNF does not play considerable role in this effect. The experience-related downscaling of inhibitory transmission might be attributed to effects gliotransmitters, most likely ATP which was shown to down-regulate GABAergic signalling acting through P2X receptors [27].

### 3.4. Role for Astrocytes in Age- and Experience-Related Changes in the Synaptic Plasticity

The above results suggest that strength of excitatory synapses can decrease with ageing whilst and inhibitory synaptic transmission can be upregulated. Potentially, these alterations can have a negative effect of long-term synaptic potentiation and, correspondingly, on learning and memory. On the other hand, the EE- and CR-induced up-scaling of glutamatergic synapses via BDNF/MSK1 cascade (Figure 4), in synergy with down-regulation of GABAergic transmission (Figure 5), could counterbalance the age-related changes in synapses and therefore have a positive impact on the long-term synaptic plasticity. 

**Figure 6:**
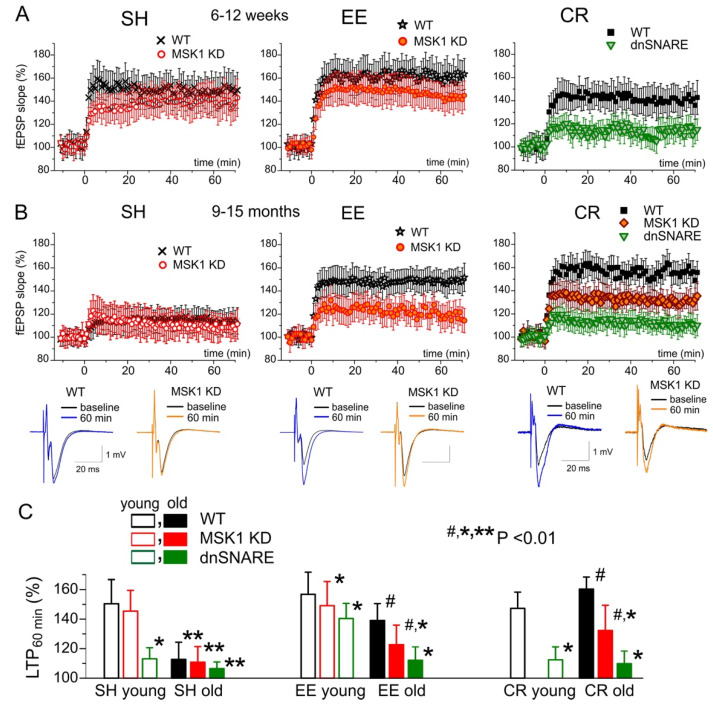
Impact of BDNF/MKS1 pathway and gliotransmission on synaptic plasticity in the neocortex. Long-term potentiation of field EPSPs (LTP) was induced in the layer 2/3 of somatosensory cortex of the wild-type, MSK1 KD and dnSNARE mice as described in the Methods section. (**A**,**B**) The time course of changes in the fEPSP induced by 50 HFS trains delivered at zero time, measured in the young (**A**) and old (**B**) mice of standard housing (SH) and mice exposed to enriched environment (EE) and caloric restriction diet (CR). Dots in the graphs represent the average of 6 consecutive fEPSPs; data are shown as mean ± SD for 7–15 experiments. Data were normalized to the fEPSP slope averaged over 10 min period prior to the HFS. The insets show the average fEPSP waveforms recorded in old mice before and 60 min after the HFS. (**C**) The pooled data on the magnitude of the long-term potentiation of the neocortical fEPSP in young and old mice of different genotypes at different conditions. Graphs show the magnitude of LTP evaluated as relative increase in the fEPSP slope at 60th min, averaged across 10 min time window and plotted against the number of HFS trains delivered. Data are shown as mean ± SD for the following number of experiments (mice used): 10–15 (7–10) for SH, 7–10 (4–5) for EE and 7–9 (4–5) for CR mice. The statistical significance (2-population unpaired *t*-test) for the difference between genotypes, age- and treatment groups was indicated by different symbols as follows: (#)—effect of EE or CR as compared to SH mice of the same genotype; (*) dnSNARE mice vs. their WT litter-mates of the same age and housing; (**) old vs. young mice of the same genotype and housing. Note the significant decrease in the LTP magnitude in old mice and significant effects of EE and CR in the old WT mice.

To verify this hypothesis, we investigated the long-term potentiation (LTP) of the field EPSPs in layer II/III of somatosensory cortex of the wild-type, MSK1 and dn-SNARE mice of two age groups (Figure 6). There was no significant difference in the LTP magnitude in the neocortex of young WT and MSK KD1 mice, both of standard housing and exposed to environmental enrichment (Figure 6A,C). However, the dnSNARE mice, both SH and EE, showed the significant deficit in the LTP as compared to their WT counterparts (Figure 6C). These results suggest that moderate (up 25–30%) decrease in the AMPA receptor-mediated signalling alone (as occurred in the MSK1 KD mice) may not be enough to cause impairment of LTP and both the decrease in the excitatory and increase in the inhibitory signalling (like those one occurring in the dnSNARE mice) are essential.

In agreement with this hypothesis, the role of BDNF/MSK1 pathway became more prominent in old age. We observed the severe deficit in the LTP in the neocortex of old WT and MSK1 KD mice of standard housing; the LTP was rescued in these mice by environmental enrichment (Figure 6). Still, the magnitude of LTP in the MSK1 KD EE mice was considerably lower (Figure 6B,C); the difference between WT and MSK1 KD mice was significant with *p* < 0.02 (Figure 6C). In the old dnSNARE mice, the EE-induced enhancement of LTP was effectively abolished (Figure 6C). Similarly to EE, CR had a prominent ‘anti-aging’ effect on the neocortical LTP which also was strongly attenuated in the MSK1 KD and dnSNARE mice. 

The differences in the effects of EE and CR on LTP between old MSK1 KD and dnSNARE mice closely agree with notion of importance of synergetic modifications in the excitatory and inhibitory plasticity: the MSK1 KD mice, where only the up-scaling of glutamatergic synapses is impaired, show smaller deficit in the LTP as compared to dnSNARE mice, where down-regulation of inhibitory synapses is impaired as well.

Combined, our data strongly support importance of astrocytic exocytosis and BDNF-mediated signalling for beneficial effects of EE and CR on synaptic transmission and plasticity in the ageing brain. 

## 4. Discussion

In our first line of experiments, we demonstrated that the long-term enhancement of astrocytic Ca^2+^ signalling can activate the same molecular cascade that was previously shown to underlie the homeostatic synaptic scaling induced by alterations in neuronal firing [9,10]. In particular, astrocyte-induced alterations in synaptic strength (Figure 1) and morphology (Figure 2) require the activity of TrkB receptors to BDNF and MSK1 kinase. Combined with the recent data, unequivocally showing the ability of astrocytes to release BDNF via exocytosis [23], our results strongly suggest that astrocyte-derived BDNF is involved in homeostatic synaptic plasticity. 

Physiological relevance of astroglial-derived BDNF and BDNF/MSK1 pathway was further substantiated by our ex vivo experiments on experience- and diet-induced alterations in synaptic signalling. We observed that exposure of mice to environmental enrichment and caloric restriction induced an increase in the glutamatergic and decrease in the GABAergic synaptic transmission (Figure 5) which converged in the facilitation of LTP induction (Figure 6). The impact of EE and CR was significantly reduced in the dnSNARE mice, indicating the critical importance of astrocytic exocytosis, which is, very likely, the main pathway of BDNF release [23]. Moreover, the effects of EE and CR on synaptic transmission and plasticity were significantly reduced in the MSK1 KD mice (Figure 4 and Figure 5), where one of the main pathways of BDNF-mediated homeostatic plasticity was impaired [9]. In parallel to alterations in synaptic function, exposure to EE and CR caused significant up-regulation of spontaneous and neurotransmitter-evoked Ca^2+^ signalling in astrocytes, which did not differ in the WT and transgenic mice. The best possible explanation for complex action of EE and CR and difference between wild-type vs. MSK1 KD and dnSNARE mice would be enhancement of Ca^2+^-dependent release of BDNF from astrocytes which, in turn, upregulates the strength of excitatory synapses via MSK1-dependent cascade. The MSK1 has been recently reported to mediate EE-induced alterations in gene expression in hippocampal neurons and enhancement of dynamic range of synapses and cognitive flexibility [12] which suggests novel putative roles for astroglia-derived BDNF in brain function.

In contrast to the excitatory synapses, experience- and diet-related plasticity of inhibitory synaptic transmission was only weakly affected by impairment of BDNF/MSK1 pathway but was critically dependent on astroglial exocytosis. These results suggest an important difference in the astroglial-driven homeostatic regulation of excitatory and inhibitory synaptic transmission: the former is modulated mainly via BDNF/MSK1 pathway whereas the latter is modulated, most likely, by other gliotransmitters. According to our previous results, the vesicular release of ATP can play an important role in astroglial-driven modulation of GABA receptors [27].

Our results revealed important trends in the age-related alterations in synaptic transmission: decrease in the postsynaptic efficacy (i.e., quantal size) of excitatory synapses and increase in efficacy of inhibitory ones and neither effect was accompanied by notable decrease in the frequency of synaptic events. The latter observation argues against a massive age-related loss of synapses because in that case one would have observed significant decrease in the mEPSCs frequency. In parallel, we observed the impairment of long-term synaptic plasticity. Combined, these results suggest that neurons retain a significant number of functional synapses and age-related cognitive decline can be associated with dysregulation rather than complete loss of synapses. Furthermore, our present (Figure 3) and previous data [15,41,42] on age-related decline in astroglial Ca^2+^ signalling and release of gliotransmitters suggest a very plausible explanation for such dysregulation of synaptic transmission—impairment of glia-driven modulation of synaptic activity. This hypothesis is strongly supported by observation that manipulations which facilitate astroglial signalling, e.g., EE, CR, reversed the changes in excitatory and inhibitory synaptic signalling back to “younger” state and rescued the long-term synaptic plasticity. The beneficial effects of EE and CR on synaptic plasticity, most likely, rely on synergetic action of BDNF and other gliotransmitters as suggested by stronger attenuation of these effects in the dnSNARE as compared to MSK1 KD mice (Figure 6). Of course, this model of age-related alterations of synaptic function needs to be explored further, in particular in relation to neurodegenerative disorders.

## 5. Conclusions

To conclude, our results strongly support the physiological importance of astroglial exocytosis, in particular the release of BDNF, for communication between astrocytes and neurons and experience-related synaptic plasticity across a lifetime.
